# Serum Levels of Commonly Detected Persistent Organic Pollutants and Per- and Polyfluoroalkyl Substances (PFASs) and Mammographic Density in Postmenopausal Women

**DOI:** 10.3390/ijerph17020606

**Published:** 2020-01-17

**Authors:** Eunjung Lee, April Kinninger, Giske Ursin, Chiuchen Tseng, Susan Hurley, Miaomiao Wang, Yunzhu Wang, June-Soo Park, Myrto Petreas, Dennis Deapen, Peggy Reynolds

**Affiliations:** 1 Department of Preventive Medicine, Keck School of Medicine, University of Southern California, Los Angeles, CA 90089, USA; 2 Cancer Registry of Norway, 0304 Oslo, Norway; 3 Department of Nutrition, University of Oslo, 0304 Oslo, Norway; 4 Department of Epidemiology and Biostatistics, University of California San Francisco, San Francisco, CA 94704, USA; 5 Environmental Chemistry Laboratory, Department of Toxic Substances Control, Berkeley, CA 94710, USA

**Keywords:** persistent organic pollutants, mammographic density, per- and polyfluoroalkyl substances, polybrominated diphenyl ethers, polychlorinated biphenyls

## Abstract

There are little epidemiological data on the impact of persistent organic pollutants (POPs) and endocrine disruptors on mammographic density (MD), a strong predictor of breast cancer. We assessed MD in 116 non-Hispanic white post-menopausal women for whom serum concentrations of 23 commonly detected chemicals including 3 polybrominated diphenyl ethers (PBDEs), 8 per- and polyfluoroalkyl substances (PFASs), and 12 polychlorinated biphenyls (PCBs) had been measured. Linear regression analyses adjusting for potential confounders were used to examine the associations between the levels of the chemical compounds, modeled as continuous and dichotomized (above/below median) variables, and square-root-transformed MD. None of the associations were statistically significant after correcting for multiple testing. Prior to correction for multiple testing, all chemicals with un-corrected *p-*values < 0.05 had regression coefficients less than zero, suggesting inverse associations between increased levels and MD, if any. The smallest *p-*value was observed for PCB-153 (regression coefficient for above-median vs. below-median levels: −0.87, un-corrected *p =* 0.008). Neither parity nor body mass index modified the associations. Our results do not support an association between higher MD and serum levels of PBDEs, PCBs, or PFASs commonly detected in postmenopausal women.

## 1. Introduction

The impact of persistent organic pollutants (POPs) on breast cancer has been of interest due to the estrogenic and endocrine disruptive properties of certain chemicals in this class [1,2], although the epidemiological evidence for this has been mixed.

Polychlorinated biphenyls (PCBs), a class of compounds consisting of over 200 different chlorinated aromatic hydrocarbons, are the most extensively studied POPs in relation to breast cancer risk. The manufacture of PCBs was banned in most countries during the 1980s. However, PCBs are still commonly detected in the general US population, both in blood [1,3] and in breast and abdominal adipose tissue [4,5]. PCBs were classified as a known human carcinogen (group 1) by the International Agency for Research on Cancer (IARC) based on sufficient evidence for carcinogenicity for malignant melanoma [2,6]. The evidence for breast cancer was considered limited. Nearly all studies investigating a summary measure of total PCBs did not find a positive association with breast cancer risk [7,8,9,10,11]. Because the endocrine-disrupting properties of PCB congeners are diverse and sometimes conflicting (e.g., weakly estrogenic, anti-estrogenic, and anti-androgenic) [1,7], others have investigated congener-specific associations. A 2016 meta-analysis of congener-specific associations suggested that certain PCB congeners (PCB-99, PCB-183, PCB-187) are associated with increased risk of breast cancer [5].

Data on other classes of POPs such as polybrominated diphenyl ethers (PBDEs) and per- and poly-fluoroalkyl substances (PFASs) remain scarce and inconsistent [12,13,14,15,16,17,18,19,20,21,22]. Five studies using post-diagnostic samples have investigated serum or adipose-level PBDEs in relation to breast cancer risk [12,14,15,16,22]. Whereas positive associations were reported for a few PBDE congeners such as BDE-47, BDE-100, and BDE-153 in an area in China highly contaminated from electronic waste recycling [12], and suggestive positive associations were reported for BDE-47 in Alaska Native women [15] and young Canadian women [22], two studies conducted among general populations in California [14,16] reported no associations. Results on PFASs have also been mixed, with studies reporting positive associations for five PFAS compounds among Greenland Inuits [20], weak positive association for a PFAS compound perfluorooctane-sulfonamide (PFOSA) and non-significant inverse association for another PFAS compound among Danish women [17], and a positive association with estrogen receptor-positive breast cancer for perfluorooctane sulfonate (PFOS) in French women [21]; in addition, no associations were found for perfluorooctanoic acid (PFOA) in two studies conducted in an area near a chemical plant [18,19], nor for six PFAS compounds commonly detected in the general population in California [13]. Little is known about the role of these chemicals with respect to pathological changes along the cancer development pathway or cancer treatment. A major limitation common to many of these studies is the use of post-diagnostic samples for cases [13,14,15,16,20,22], with some studies using post-treatment samples [13,14].

Mammographic density (MD) is a measure of the relative amounts of epithelium and stroma in the breast and is one of the strongest known predictors of breast cancer risk [23,24,25]. Compared to women with little or no density (<5%), women with extremely dense breasts (>75%) are at 4–5 times increased risk of breast cancer [25]. MD has been associated with established risk factors of breast cancer such as having fewer children, later menopause, and receiving estrogen and progestin combined hormone therapy [26,27,28]. For these reasons, MD has been used as an early marker of breast cancer in clinical trials [29,30] and etiological studies of breast cancer [31,32,33,34,35,36]. A cross-sectional study investigating the associations between breast cancer risk factors and MD can provide useful insights into their roles in breast cancer [31,32]. We investigated the association between serum levels of POPs and MD, a strong risk factor for breast cancer, using data from control participants in a breast cancer case–control study nested in the California Teachers Study (CTS) [13].

## 2. Materials and Methods

Study Participants: Study participants were drawn from control participants of a breast cancer case–control study nested in the CTS [13]. The CTS is a prospective cohort of 133,479 female California public school professionals who returned a mailed questionnaire in 1995–1996 and provided information on various breast cancer risk factors [37]. The case–control substudy comprised 902 invasive breast cancer cases diagnosed between 1 January 2006 and 1 August 2014 aged less than 80 years at diagnosis and 858 controls drawn from a probability sample of at-risk CTS participants frequency-matched to cases by age at baseline (5-year age groups), race/ethnicity, and the region of residence (regional cancer registry of California) [13]. These substudy participants completed an interview-administered questionnaire and provided blood samples between October 2011 and August 2015. For the current study, with a target sample size of 160, we sent a study invitation letter along with a consent form, a Health Insurance Portability and Accountability Act (HIPAA) authorization form, and a survey to update information on breast cancer risk factors to 254 women selected from 331 postmenopausal substudy participants between the ages of 60 and 80 years, whose laboratory assays were completed or in process (i.e., samples transported to the laboratory) as of July 2015. Of the 331 women, all 83 nulliparous and 171 of the 248 parous women were invited. Nulliparous women were over-sampled because we were particularly interested in identifying environmental risk factors in this at-risk group. Non-respondents were contacted by a follow-up mail and up to two telephone calls. After excluding 22 women who did not have mammograms taken within 5 years, 155 women (67%) participated during the recruitment phase between January 2015 and August 2015. Reasons for non-participation were refusals (n = 19) and no response during the recruitment phase (*n* = 57).

Data collection and MD assessment: Participants mailed back a signed informed consent, an HIPAA form, and a completed questionnaire on important covariates such as height, weight (current, 5 years ago, 10 years ago), menopausal status, hormone therapy (HT), location and year of recent mammogram screenings. Mammograms were collected for all 155 women. After excluding 15 mammograms presenting technical difficulties, one of the authors (GU) assessed MD for 140 mammograms using the USC Madena software, a validated computer-assisted method to quantitatively assess MD [30,38,39]. In brief, GU assessed the absolute density of each mammogram, and a research assistant trained by EL assessed the total area of the breast. MD was calculated as the percentage of the absolute density divided by the total area of the breast. Reader reproducibility was excellent (r = 0.98; 37 random duplicates). All subjects gave their informed consent for inclusion before they participated in the study. The study was conducted in accordance with the Declaration of Helsinki, and the protocol was approved by the Institutional Review Boards at the Cancer Prevention Institute of California (2010-017), the State of California Health and Human Services Agency (12-09-0732), the University of Southern California (HS-14-00627), and the University of California San Francisco (18-25344).

Laboratory measurements: Blood samples were processed within hours of collection, and the separated serum stored at −20 °C until analysis. Laboratory methods for the measurement of serum levels of PBDEs, PFASs, and PCBs were described previously [13,16,40]. Briefly, PCBs and PBDEs were measured using automated solid-phase extraction (SPE) and gas chromatography (GC)/high-resolution mass spectrometry (HRMS); PFASs were measured using an online SPE–liquid chromatography–tandem MS (SPE–LC–MS/MS) method. PCB and PBDE chemical levels were lipid-normalized and expressed in units of ng/g lipid. For results below the laboratory limit of detections (LODs), we used LOD/$\sqrt{2}$. In the statistical analyses, we only included the 23 chemicals with detection frequency of 75% or higher: 3 PBDEs, 8 PFASs, and 12 PCBs (Table S1).

Statistical Analysis: Analyses were restricted to 116 non-Hispanic white women due to the small numbers for each of the other race/ethnic subgroups (African Americans, Hispanics, Asians/Pacific Islanders, unknown race). The association between each chemical (above vs. below median) and square-root-transformed MD was examined using multivariable linear regression, adjusting for age, body mass index (BMI) (kg/m^2^), parity (0, 1−2, 3+, non-full-term pregnancy), and estrogen–progestin combined hormone therapy use (never, former, current) at the time of mammography. Additional analyses that adjusted for the type (manufacturer) of the mammography systems (Hologic, GE, other), modeled the serum levels as continuous variables, and used untransformed MD did not change the results; thus, these variables were not used in the final model. We conducted stratified analyses by parity, BMI (≥25 vs. <25 kg/m^2^), and estrogen–progestin combined hormone therapy (never or ever used) and calculated *p* values for the interactions by introducing product terms and conducting Wald tests. The *p*-values were corrected for multiple testing using the Bonferroni method.

## 3. Results

The characteristics of the study participants are presented in Table 1. Mean age at mammogram and mean age at blood draw were 69.2 years and 67.9 years, respectively. Only 7% of participants were using estrogen–progestin combined hormone therapy at the time of mammogram. For approximately half of the participants (47%), the collected mammogram was taken within one year of blood draw.

Of the 23 chemicals analyzed, the smallest *p-*values were observed for PCBs 153 (*p =* 0.008) and 138 (*p =* 0.010), but these associations did not remain statistically significant after Bonferroni correction (Table 2 and Table S1). We did not observe significant associations in subgroups by parity, BMI, or estrogen–progestin combined hormone therapy (EPT) use, or evidence of effect modification. The regression coefficients for all chemicals with un-corrected *p-*values < 0.05 were less than zero, suggesting inverse associations between their increased levels and MD, if any. For example, in the overall analyses, only PCB-138 and PCB-153 were associated with MD with a *p-*value < 0.05 before correcting for multiple testing. The regression coefficients observed for PCB-138 and PCB-153, i.e., −0.81 and −0.87, respectively, indicate that women with above-median concentrations of these two chemicals had a similar but slightly lower MD compared to women with below-median concentrations. Similarly, in the subgroup-specific analysis among parous women, the regression coefficients for the two chemicals associated with a *p-*value < 0.05 before multiple testing correction, namely, PCB-203 and PCB-74, were also less than zero (−0.78 and −0.99), indicating an inverse association, if any. All *p-*values for the interactions with parity, BMI, or EPT use were >0.05 before correcting for multiple testing.

## 4. Discussion

MD is a strong risk factor for breast cancer and increases in response to exogenous hormone use in postmenopausal women [30,38]. Despite biological plausibility, our results in postmenopausal women provide little evidence of associations between serum levels of PBDEs, PFASs, or PCBs and MD. Only one prior study has investigated the association between PCBs and MD. Among 106 post-menopausal women in Canada, 21 of the 24 tested PCB congeners were not associated with MD, and the other 3 congeners (PCB-153, 183, and 196) showed inverse associations, with *p-*values of 0.03, 0.004, 0.04 (not corrected for multiple testing), which are directionally consistent with our results [41]. PBDEs and PFASs have not been investigated in relation to MD.

Whereas the data on MD are sparse, substances in these chemical families (PBDEs, PFASs, PCBs) have been studied with respect to breast cancer risk. Results on PCBs in relation to breast cancer risk are inconsistent with the null findings in relation to MD ([41] and the current study). In a congener-specific meta-analysis, PCB-99, PCB-183, and PCB-187 were associated with increased risk of breast cancer, although the data were from a relatively small number of case–control studies [5]. It is possible that these PCBs increase breast cancer risk through a mechanism other than increasing MD.

Previous studies on PBDEs have been limited and reported mixed findings. Although one study of the adipose levels of PBDEs in a contaminated area in China reported a positive association for BDE-47, BDE-100, and BDE-153 [12], these associations were not observed in the general population in California [16] or in studies using serum samples in the CTS [14] or among Native Alaskan women [15]. A recent study of young (age <45 years) Canadian women, the Ontario Environment and Health Study, reported a positive association for BDE-47, which was statistically significant only among premenopausal women (RR = 1.73, 95% CI = 1.02–2.94) [22]. In other studies, more than half of case patients were postmenopausal (~55% in [12,15,16]; ~95% in [14]). Studies reporting no associations [14,15,16] did not separately present results among premenopausal women; however, the sample sizes for premenopausal women in these studies were limited (N of premenopausal case patients ranged from 29 to 43) [14,15,16]. Each of these studies has several limitations, including modest sample sizes (number of case patients ranging from 75 to 209) [12,15,16], the fact of being hospital-based case–control studies that comprised control participants with benign breast disease [12,15,16] or diseases unrelated to breast cancer and who underwent surgery [12], which raises concerns for over-matching, or the fact of relying on post-diagnostic samples of breast cancer patients [12,14,15,16,22]. Case patients from the Ontario Environment and Healthy Study (n = 305) were identified from the Ontario Cancer Registry, thus many of these samples are likely to have been collected after cancer treatment [22].

Samples from the CTS study which had the largest sample sizes (n = 902 case patients) were collected on average 35 months after diagnosis and treatment [14]. Although our group did not observe any indication of changes in PBDE levels according to the time interval between diagnosis and sample collection [14], it remains unknown whether breast cancer treatment impacts PBDE levels [14]. Results from a prospective study using pre-diagnostic samples will provide additional insights into the effects of PBDEs. Alternatively, it is also possible that PBDEs increase breast cancer risk in young premenopausal women [22] or in women living in highly contaminated areas [12]. Nonetheless, our null findings provide support that PBDEs may not be strong risk factors of breast cancer among postmenopausal women in the general population.

Similarly, PFASs were not, or were at most inversely, associated with breast cancer risk in the CTS [13]. The only published prospective study of diverse classes of PFASs (16 PFASs) used serum samples collected during pregnancy in Danish women and also reported no associations or non-significant inverse associations between those PFASs and premenopausal breast cancer risk, except for a weak positive association observed for perfluorooctane-sulfonamide (PFOSA) [17]. Another prospective study nested in the French E3N (Etude Epidémiologique auprès de femmes de la Mutuelle Générale de l’Education Nationale) cohort investigated two compounds, PFOS and PFOA, and did not observe significant associations with overall breast cancer; however, there was a significant positive linear association between one of the compounds (PFOS) and the risk of estrogen receptor-positive subtype of breast cancer [21]. PFOS levels in the E3N study were much higher than the levels in the CTS (median levels among controls: 17.3 ng/mL and 6.95 ng/mL, respectively) [21]. PFOA was also investigated in two studies in a contaminated area in North Carolina using an ecological study design [19] or mathematically estimated PFOA levels [18], both reporting no associations [18,19]. These findings are in contrast to significant positive associations reported among Greenland Inuits for a summed concentration of 16 PFASs as well as 5 individual PFAS compounds including PFOA, perfluorononanoic acid (PFNA), perfluorodecanoic acid (PFDA), perfluorohexane sulfonic acid (PFHxS), and perfluorooctane sulfonic acid (PFOS) [20]. The median serum levels of some (PFNA, PFDA, and PFOS), although not all (PFOA and PFHxS), of these PFAS compounds were much higher (~2 fold to ~5 fold) among the Greenland Inuits control women compared to the levels in the CTS [13]. This may have contributed to the conflicting findings. In particular, the median level of PFOS in Inuits was 18.2 ng/mL, similar to the level in the E3N study (17.3 ng/mL) [21]. Effect modification by polymorphisms in genes involved in estrogen metabolism such as *CYP17A1* -34T/C (rs743572) and *COMT* Val158Met (rs4680) has been proposed [42,43]; however, the modifying effects of these polymorphisms were not consistent across PFAS compounds or across studies, and frequencies of alleles potentially associated with increased susceptibility to PFASs among Inuits [42] were only slightly higher (rs4680 Met allele) or even lower (rs743572 T allele) in Inuits than in European women [42,44]. Taken together, findings from these studies and our own study suggest that PFAS compounds do not substantially increase breast cancer risk or MD, at least in postmenopausal women. Reasons for the discrepancies in Inuit women as well as a positive finding for PFOSA exposure during pregnancy in Danish women [17] warrant further study.

Our study has several limitations, and our results need to be interpreted with caution. Our chemical measurements were based on blood samples collected at approximately the same times of the outcome (MD) measurement and were restricted to postmenopausal women. Therefore, the measured chemical levels may not represent etiologically relevant exposure. Our study cannot address the question whether elevated levels of these chemicals in earlier time periods with different hormonal milieux, such as childhood, puberty, premenopausal period or menopausal transition, are associated with MD and with breast cancer risk. Additional studies in younger women need to be conducted before excluding POPs and PFASs as risk factors in the general population. The suggestion that POPs may cause reproductive abnormalities in women [45], which is related to breast cancer risk, should also be further studied. Our results are also limited by the relatively small sample size and the inclusion of only non-Hispanic white women. However, our statistical power is similar to or greater than that of the only other study on PCBs with a similar sample size (n = 106) [41], and no studies have been conducted on PBDEs and PFASs in relation to MD.

## 5. Conclusions

Findings from our cross-sectional study do not support a positive association between PCBs, PBDEs, and PFASs and MD among postmenopausal women.

## Figures and Tables

**Table 1:** Characteristics of 116 postmenopausal non-Hispanic white participants included in this study.

Characteristics	Mean ± SD	N (%)
Age at Mammogram (Years)	69.2 ± 4.9	
Age at Blood Draw (Years)	67.9 ± 4.9	
Body Mass Index (BMI) at Mammogram (kg/m^2^)	25.3 ± 4.8	
Mammographic Density (Percent)	14.1 ± 13.1	
Estrogen–progestin Combined Hormone Therapy at Mammogram		
Never		74 (64%)
Former Use		34 (29%)
Current Use		8 (7%)
Total Number of Births		
0		37 (32%)
1−2		58 (50%)
3+		21 (18%)
Time Interval between Mammogram and Blood Draw		
<1 year		54 (47%)
1 to <2 years		35 (30%)
2 to <3 years		19 (16%)
3 to <4 years		8 (7%)

**Table 2:** Association between square-root-transformed mammographic density (%) and chemicals in all participants and in any of the subgroups based on parity and BMI. Results are presented for chemicals with a *p-*value < 0.05 in any of the subgroup or overall analyses before correcting for multiple testing *.

Chemical	All (n = 116)		Parous (n = 80)		Nulliparous (n = 36)		BMI (<25 kg/m^2^) (n = 56)		BMI (≥25 kg/m^2^) (n = 60)	
	Beta ^¶^ (SE)	P_adj_^†^ (*p*) ^§^	Beta ^¶^ (SE)	P_adj_^†^ (*p*) ^§^	Beta ^¶^ (SE)	P_adj_^†^ (*p*) ^§^	Beta ^¶^ (SE)	P_adj_^†^ (*p*) ^§^	Beta ^¶^ (SE)	P_adj_^†^ (*p*) ^§^
PCB−138	−0.81 (0.31)	0.22 (0.010)	−0.78 (0.41)	>0.99 (0.060)	−0.94 (0.50)	>0.99 (0.068)	−1.02 (0.39)	0.26 (0.011)	−0.33 (0.49)	>0.99 (0.50)
PCB−153	−0.87 (0.32)	0.18 (0.008)	−0.79 (0.41)	>0.99 (0.059)	−1.10 (0.55)	>0.99 (0.053)	−0.58 (0.41)	>0.99 (0.17)	−1.22 (0.49)	0.38 (0.016)
PCB−203	−0.60 (0.32)	>0.99 (0.062)	−0.78 (0.39)	>0.99 (0.049)	−0.08 (0.57)	>0.99 (0.90)	−0.12 (0.41)	>0.99 (0.77)	−1.12 (0.48)	0.55 (0.024)
PCB−74	−0.53 (0.33)	>0.99 (0.11)	−0.99 (0.42)	>0.99 (0.020)	0.32 (0.55)	>0.99 (0.56)	−0.75 (0.43)	>0.99 (0.084)	−0.23 (0.49)	>0.99 (0.64)
PCB−187	−0.45 (0.32)	>0.99 (0.16)	−0.38 (0.42)	>0.99 (0.37)	−0.67 (0.56)	>0.99 (0.24)	−0.01 (0.44)	>0.99 (0.97)	−1.12 (0.46)	0.43 (0.019)

* All analyses were adjusted for age (continuous), BMI (continuous), parity (0, 1−2, 3+), and estrogen–progestin combined hormone therapy use (never, former, current). ^¶^ Linear regression coefficient representing differences in √ mammographic density for women with each chemical level above median vs. below median. ^†^ *p* values corrected for multiple testing using the Bonferroni method; ^§^ *p* values not corrected for multiple testing.

## Supplementary Materials

The following are available online at https://www.mdpi.com/1660-4601/17/2/606/s1. Supplementary Table (Table S1): Associations between each chemical (above vs. median) and square-root-transformed percent mammographic density (PMD) in all women (n = 116) and in women divided by parity, BMI, estrogen–progestin combined hormone therapy use.
